# Reconciling the opposing effects of warming on phytoplankton biomass in 188 large lakes

**DOI:** 10.1038/s41598-017-11167-3

**Published:** 2017-09-07

**Authors:** Benjamin M. Kraemer, Thomas Mehner, Rita Adrian

**Affiliations:** IGB Leibniz-Institute of Freshwater Ecology and Inland Fisheries, Berlin, 12587 Germany

## Abstract

Lake ecosystems are deeply integrated into local and regional economies through recreation, tourism, and as sources of food and drinking water. Shifts in lake phytoplankton biomass, which are mediated by climate warming will alter these benefits with potential cascading effects on human well-being. The metabolic theory of ecology suggests that warming reduces lake phytoplankton biomass as basal metabolic costs increase, but this hypothesis has not been tested at the global scale. We use satellite-based estimates of lake surface temperature (LST) and lake surface chlorophyll-a concentration (chl-a; as a proxy for phytoplankton biomass) in 188 of the world’s largest lakes from 2002-2016 to test for interannual associations between chl-a and LST. In contrast to predictions from metabolic ecology, we found that LST and chl-a were positively correlated in 46% of lakes (p < 0.05). The associations between LST and chl-a depended on lake trophic state; warming tended to increase chl-a in phytoplankton-rich lakes and decrease chl-a in phytoplankton-poor lakes. We attribute the opposing responses of chl-a to LST to the effects of temperature on trophic interactions, and the availability of resources to phytoplankton. These patterns provide insights into how climate warming alters lake ecosystems on which millions of people depend for their livelihoods.

## Introduction

Metabolic ecology has gained prominence, in part, for its capacity to explain and predict macroecological patterns and the influences of climate warming on the Earth. Based on the fundamentals of metabolic ecology, warming increases phytoplankton’s demand for resources to support higher metabolic rates at higher temperature^1^. If the availability of rate-limiting resources remains constant as lake ecosystems warm, resources will become scarcer relative to their demand causing a metabolic deficit^2, 3^. Metabolic deficits at higher temperatures would leave lakes capable of supporting less phytoplankton biomass^2, 3^. This theory has been used to explain how lake phytoplankton sizes and abundances decrease with warming in freshwater mesocosms^2, 4^.

However, warming has the potential to affect trophic interactions, and the availability of resources for phytoplankton, which could alleviate^5^ or exacerbate^6^ the warming-induced metabolic deficit. Thus, the cumulative effects of warming on phytoplankton biomass remain uncertain for most of the world’s global population of large lakes. Uncertainty in the simple directionality of responses of phytoplankton biomass to temperature prevents meaningful estimates of how climate warming will affect lake carbon cycling^7^, food webs^8^, and biodiversity^8–10^ at the global scale. Understanding which lake attributes are associated with the strongest positive and negative effects of temperature on phytoplankton biomass could guide future research and inform lake management.

To address this uncertainty, we compared interannual variability in surface chl-a to LST for 188 of the world’s largest lakes (listed in Supplementary Table 1). While they make up only a small proportion of Earth’s lakes, these 188 lakes contain much of Earth’s liquid surface freshwater^11^, lake surface area^11^, and endemic lake species^12^. Even if the 188 lakes are biased relative to the global population of lakes in terms of their surface area, they are representative in terms of their depth, elevation, latitude, temperature, and average phytoplankton biomass. Changes in phytoplankton biomass in some large lakes have been shown to have substantial effects on species, economies, and livelihoods^6, 13^.

Satellite measurements of lake colour provide a useful, high-resolution (spatial and temporal) phytoplankton biomass proxy that enables links to LST at broad spatial scales^14^. We obtained daily chl-a concentration data from 2002-2016 from the Moderate Resolution Imaging Spectroradiometer (MODIS)-Aqua mission as pre-processed by the National Aeronautics and Space Administration’s (NASA) Ocean Biology Processing Group (OBPG). We merged the chl-a data with daily LST data from 2002-2016 from the Group for High Resolution Sea Surface Temperature (GHRSST) (3,340,741 coincident observations). We calculated average lake-wide Kendall’s rank correlations between chl-a and LST after accounting for seasonal and spatial variability within lakes (see methods). The average lake-wide correlations reported here reflect interannual associations between chl-a and LST, not seasonal associations, and are thus most likely to reflect the directionality of long-term lake temperature forcing. *In situ* monitoring data from the North American Great Lakes were used to validate the chl-a and LST data (see methods).

## Results and Discussion

**Figure 1:**
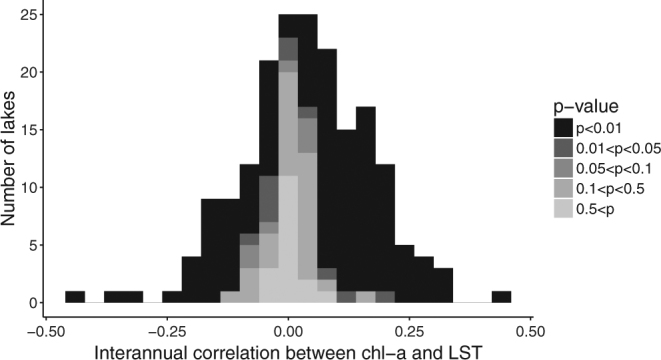
Stacked bar density distribution of lake-wide, interannual, Kendall’s rank correlation coefficients between chl-a and LST for 188 of the world’s largest lakes. The significance of the p-value associated with the coefficient is indicated by the greyscale. Correlations between chl-a and LST are highly variable across lakes (i.e. both positive and negative correlations) and strong (i.e. high proportion of correlation coefficients are significant, Wilcox signed-rank test, p < 0.05).

**Figure 2:**
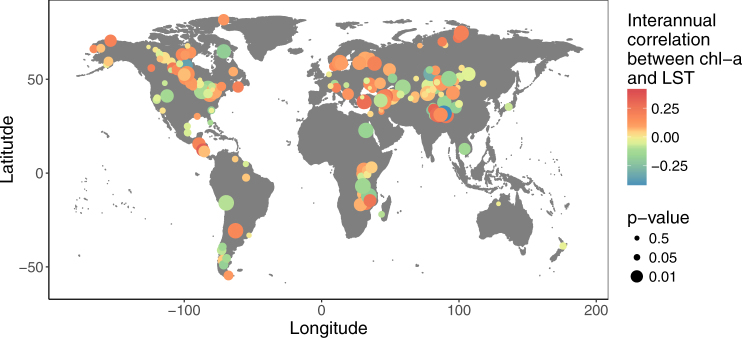
Regional variability in the lake-wide, interannual, Kendall’s rank correlation coefficients between chl-a and LST for 188 of the world’s largest lakes. The size of the dot is proportional to the significance of the correlation. The color of the dot reflects the magnitude of the correlation. The map was made using R version 3.3.3^55^ (https://www.R-project.org). Land mass polygons are from an open-access digitial map in the package “ggmap” (https://github.com/dkahle/ggmap).

We found that the lake-wide average correlations between chl-a and LST were highly variable across lakes (i.e. both positive and negative correlations) and a high proportion of correlation coefficients were significant (Wilcoxon signed-rank test, p < 0.05; Figs 1 and 2). Our analysis showed that 38% of the lakes had negative correlations between chl-a and LST (72 out of 188 lakes), of which 68% (49 lakes) were significant after correcting for multiple comparisons (Wilcoxon signed-rank test, *p* < 0.05; Fig. 1). These negative interannual correlations between chl-a and LST may, in part, reflect reductions in phytoplankton size and abundance with warming as predicted from metabolic theory^15^.

Despite the metabolic deficit imposed on phytoplankton by warming^2^, lake-wide correlations between chl-a and LST were positive in 62% of lakes (116 out of 188), of which 74% (86 lakes) were significant after correcting for multiple comparisons (Wilcoxon signed-rank test, *p* < 0.05; Fig. 1). Thus, our overall findings did not offer strong support for predictions from the metabolic theory that warming will reduce phytoplankton biomass.

To offer insights about which lake attributes influence the association between chl-a and LST, we compared the average lake-wide correlations between chl-a and LST across lakes to their ecological, morphometric, and geographical characteristics. To do this, we used boosted regression trees to determine which of eight lake characteristics (elevation, latitude, lake surface area, lake perimeter, mean depth, salinity, median temperature, and median chl-a) best explained variability in lake-wide average correlations between chl-a and LST.

**Figure 3:**
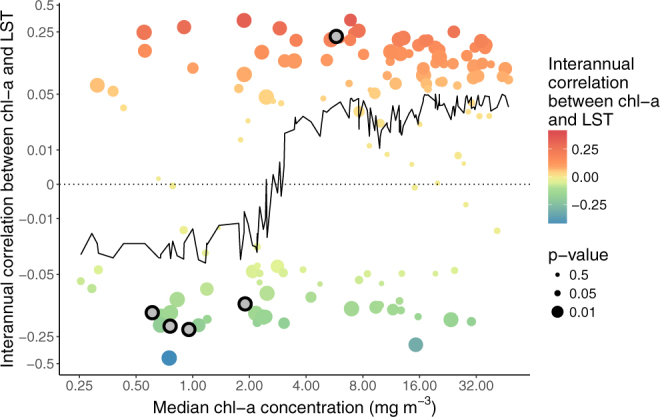
Each coloured dot represents one lake’s average lake-wide correlation coefficient between chl-a and LST, as in Fig. 2. The size of the dot reflects the significance of the Kendall’s rank correlation p-value. The colour of the dot reflects the value of the correlation coefficient in parallel with the y axis. The y-axis is hyperbolic sine transformed and the x axis is log_10_ transformed for visualization purposes. The grey dots represent the interannual correlation coefficients for the five North American Great Lakes based on *in situ* data. The black line represents the effect of median chl-a concentration on the interannual correlation between chl-a and LST using remotely-sensed data after accounting for and removing variability attributable to other lake characteristics (elevation, latitude, lake surface area, lake perimeter, mean depth, salinity, median temperature; see methods).

We found that lakes with relatively low median chl-a tended to have more negative correlations between chl-a and LST whereas lakes with relatively high median chl-a tend to have more positive correlations (Fig. 3). This pattern was also true for the North American Great Lakes using *in situ* data collected by the EPA (Fig. 3). Thus, lake warming tended to amplify lake-to-lake variability in phytoplankton biomass whereby phytoplankton-poor lakes were poorer in warm years and phytoplankton-rich lakes were richer in warm years. Lake median chl-a had the highest “relative influence” on the correlation between LST and chl-a (29%) in the boosted regression trees, where “relative influence” is a function of the frequency with which a variable was selected for inclusion in each iterated regression tree and the improvement to the model that resulted from its inclusion.

Our results are consistent with previous work which suggests that the effect of warming on the trophic transfer efficiency of phytoplankton biomass to higher trophic levels depends on lake trophic state^8, 16, 17^. We use the term “trophic state” to refer to the continuous gradient between phytoplankton poor-lakes (chl-a < ~3 μg/L) and phytoplankton-rich lakes (chl-a > ~20 μg/L). In phytoplankton-rich lakes, lake warming strongly favours phytoplankton species such as cyanobacteria which are less efficiently consumed by grazers^8, 18, 19^. The resulting reductions in trophic transfer efficiency from phytoplankton to their consumers may lead to an accumulation of phytoplankton biomass in warm years in phytoplankton-rich lakes^20^. However, community shifts toward less edible phytoplankton species are weaker in phytoplankton-poor lakes because cyanobacteria abundance in phytoplankton-poor lakes depends more on nutrient availability than on temperature alone^18, 20, 21^.

Our results also support previous work which suggests that phytoplankton responses to warming depend on trophic state due to the constraints of lake trophic structure. Phytoplankton-rich lakes tend to have strong populations of zooplanktivorous fishes leading to “grazer-release”^22^ on phytoplankton^23–25^. Warming is expected to increase consumption rates, which would strengthen “grazer release” and promote higher phytoplankton biomass^23–25^. In contrast, where piscivores are present, chl-a may be low because of high grazing rates by primary consumers^23–25^. Consequently, warming induced enhancement of consumption rates would have the opposite effect of further reducing phytoplankton biomass in phytoplankton-poor lakes^8, 26, 27^. Thus, variability in the effect of warming on top-down limitation of phytoplankton biomass may partially explain why the correlations between chl-a and LST depended on lake trophic state (Fig. 3).

The effects of warming on lake nutrient budgets will also depend on lake trophic state^10^. Negative correlations between chl-a and LST may be most common in phytoplankton-poor lakes because surface warming enhances thermal stratification which can trap nutrients below the photic zone where they are unavailable to surface phytoplankton^6^. This effect is most pronounced in phytoplankton-poor lakes because internal nutrient loading via vertical mixing is often the primary source of nutrients to phytoplankton there^28–30^. In contrast, external nutrient inputs and their subsequent recycling tend to dominate nutrient budgets in phytoplankton-rich lakes^20, 28, 31^. External nutrient inputs may increase with warming driven by climate-mediated shifts in rainfall and land use^20, 32^, which would elicit more positive correlations between chl-a and LST in phytoplankton-rich lakes. However, these effects are likely to be region-specific^30^ and whether climate-mediated shifts in rainfall and land use will increase or decrease phytoplankton biomass at the global scale remains uncertain^30^.

Overall, we found that warming amplifies lake trophic states, but some lakes diverged from this pattern (Fig. 3). These lakes may be most affected by mechanisms linking chl-a and LST which act regardless of lake trophic state. For instance, positive correlations between chl-a and LST may arise from the expansion of the growing season irrespective of lake trophic state. Lake surface warming can expand the duration of thermal stratification which prevents phytoplankton from sinking to the aphotic zone where phytoplankton are light limited^33^. By alleviating light limitation, warming expands the growing season, thereby increasing phytoplankton biomass. Such cases would elicit positive correlations between chl-a and LST on interannual timescales regardless of lake trophic state. Metabolic deficits are expected to act on all lakes which could elicit negative relationships between chl-a and LST. However, the deviations from that prediction observed here suggest the need to incorporate resource availability, species composition, and trophic interactions into metabolic theory so that it may better predict global lake responses to climate change^34^. In summary, the relationship between chl-a and LST reflects a combination of indirect and direct effects, only some of which would be expected to vary along the lake trophic state gradient.

Our results are corroborated by *in situ* data and paleo-proxies from the North American Great Lakes (see Supplementary Figures 1–4 and Supplementary Table 2) and the African Great Lakes^6, 35^ which show long-term trends in phytoplankton biomass which match those in our study. The patterns we saw across lakes are also paralleled in the global oceans. Negative relationships between chl-a and sea surface temperature (SST) are common in the phytoplankton-poor regions of the tropical oceans^36^. Whereas the interannual correlation between chl-a and SST is more positive in the polar, relatively phytoplankton-rich regions of the marine environment^37^. As we have demonstrated for the world’s large lakes, these are not paradoxical responses because the indirect effects of temperature on phytoplankton in different contexts explain why opposite responses to warming can occur within the same ecosystem type.

We present these results with several caveats. While most lake-wide correlations in our results were significant, most interannual associations between chl-a and LST were weak (Figs 1–3). This reflects what we already know—that temperature is important, but only one of many factors influencing phytoplankton biomass. Time lags between temperature changes and its effects on phytoplankton biomass may also weaken the correlations between LST and chl-a which reflect only temporally coincident effects. We used the correlation coefficients calculated here as indicators of the directionality of surface phytoplankton biomass responses to temperature, but they conceal the magnitude of those responses. To determine the magnitude of phytoplankton biomass responses to temperature will require improved calibration procedures for globally distributed individual lakes which are not currently available^38^. The simple band algorithms used here to convert lake colour data to chlorophyll-a concentration have been updated and improved in recent years^39^, but have not been calibrated for individual lakes. The optical properties of inland waters can make it difficult to use simple band algorithms to distinguish between chlorophyll-a and other dissolved and particulate substances (e.g. coloured dissolved organic matter)^40, 41^. The development and validation of algorithms for optically complex waters could be substantially improved through rigorous validation against *in situ* data across the full spectrum of inland water types. However, research groups currently have access to *in situ* data from only a limited range of lakes. Regardless, our validation against *in situ* data from the North American Great Lakes suggests that our results may be robust to deviations between satellite and *in situ* data (see Supplementary Figures 1–4 and Supplementary Table 2).

Another important caveat is that our results reflect patterns at the surfaces of lakes during ice-free and cloud-free periods only. The relationship between chl-a and LST may vary with depth, ice cover, and cloud cover in ways that diverge from patterns shown here. While we have considered phytoplankton biomass as a response to temperature throughout our manuscript, phytoplankton sizes and abundances can also influence water temperature through its effect on light penetration in the water column^42^. The relationships between chl-a and LST found in specific lakes could be confounded by the influence of an independent variable (i.e. wind, water level, species invasions) which could elicit spurious correlations. Thus, we urge caution in interpreting the correlation coefficient in any specific lake as a strict warming effect. However, these independent drivers of both temperature and chl-a are likely to be lake-specific, thus we doubt that they would consistently bias our results comparing across many globally distributed lakes.

Our results may apply broadly to the global population of lakes despite our results being based on a subset of only the largest lakes, because lake surface area did not have a strong influence on its correlation between chl-a and LST. The relative influence of surface area in the boosted regression trees was only 9% which is less than the null expectation given eight variables in the model (12.5%). However, according to the boosted regression trees, smaller lakes did have slightly more positive correlation coefficients between chl-a and LST than larger lakes. Extrapolating this trend to smaller lakes would indicate that chl-a and LST may be more positively correlated on average for the complete global population of lakes. Future work using lake colour data from satellites with finer spatial resolution (e.g. Sentinel-3A) should enable direct tests of how warming affects phytoplankton biomass in smaller lakes. Merged chl-a data products which combine measurements from multiple sensors may also enhance the spatial and temporal coverage for smaller lakes, enabling the inclusion of more lakes in future studies. Our computational approach also required more data per lake than other less computationally intensive approaches would have required. Simpler approaches using linear models instead of boosted regression trees could increase the number of lakes with sufficient data for model fitting.

Changes in phytoplankton biomass that result from lake warming are likely to affect lake carbon cycles^7^, lake warming rates^43^, lake ecology^44, 45^, and lake-derived benefits to humans^46^. Higher phytoplankton biomass in phytoplankton-rich lakes may exacerbate problems associated with anthropogenic lake nutrient enrichment, such as the expansion of anoxic zones, harmful algal blooms, fish die-offs, and reduced water clarity. Managers may need to reduce anthropogenic nutrient loads that were acceptable in the past to maintain ecosystem functions in phytoplankton-rich lakes as they warm^47, 48^. In contrast, the reduction of phytoplankton biomass in phytoplankton-poor lakes with warming presents its own potential management challenges such as reduced fisheries productivity^6, 45^. For instance, fish production in Lake Tanganyika has already been substantially diminished as a result of climate-mediated reductions in phytoplankton biomass and production^6^. In some cases, managers may want to fertilize lakes to sustain fish production^45^, but managers must prudently weigh the changing costs and ecological risks of those actions. Thus, the amplification of lake trophic states with warming may require adaptive lake management efforts at the local level to prevent loss of lake benefits to humans. Otherwise, human livelihoods are likely to be affected across gradients in lake trophic state at the global scale.

## Methods

### Data extraction

Daily estimates of lake surface temperature were obtained from the blended, level-4 data product of the Group for High Resolution Sea Surface Temperature (GHRSST) version 4.1^49^. GHRSST data at 1 km resolution are based upon night time skin and sub-skin surface temperature observations from the NASA Advanced Microwave Scanning Radiometer-EOS (AMSRE), the Moderate Resolution Imaging Spectroradiometer (MODIS) on the NASA Aqua and Terra platforms, the US Navy microwave WindSat radiometer, Advanced Very High Resolution Radiometer (AVHRR) on several National Oceanic and Atmospheric Administration (NOAA) satellites, and *in situ* SST observations from NOAA. Daily estimates of lake surface chl-a were obtained at 4 km resolution from the MODIS Aqua mission dataset processed to Level-3 data by the Ocean Biology Processing Group^50^ of NASA. Chl-a data are generated using the OCI band ratio algorithm based on the method of Hu *et al*.^39^. Chl-a and LST data covered the time period from July 2002 to November 2016.

To ease the computational intensity of this work, we used 0.1-degree median-filtering followed by data subsetting to a spatial resolution of 0.1-degrees for all analyses. We also used a 5-day median filter followed by data subsetting to a temporal resolution of 5 days for all analyses. Lake sections were identified using the level 1 Global Lakes and Wetlands Database (GLWD-1) comprising the 3067 largest lakes and 654 largest reservoirs worldwide including basic attribute data (surface area, perimeter, elevation, etc.)^51^. Satellite data were available from only a subset of these water bodies because many lakes were too small to be included or were persistently obstructed by clouds or ice.

### Modelling approach

To estimate the average lake-wide correlation coefficient between chl-a and LST (*r*_*lake*_), we first calculated correlation coefficients separately for every latitude-longitude-day of the year combination in our dataset. To do this, we subdivided all observations in the 15-year time series by its pixel (resolution of 0.1 degrees). We further subdivided the data from each pixel by the day of the year on which the data were measured. As a result, each subdivision was composed of up to 15 observations of chl-a and LST—one observation for each of the 15 years in the time series. For subdivisions with at least eight coincident observations of chl-a and LST (following minimum sample size recommendations from published literature^52^), we used non-parametric Kendall’s rank correlation to determine the interannual relationship between chl-a and LST (312,963 correlation coefficients in total). We used non-parametric Kendall’s rank correlations to assess the relationship between LST and chl-a to avoid spurious correlation coefficients in cases where the relationship between *in situ* and satellite data are nonlinear or where the linear slope was not equal to one.

We calculated each lake’s average lake-wide correlation coefficient between chl-a and LST (*r*_*lake*_) using the correlation coefficients calculated for every latitude-longitude-day of the year combination (*r*_*i*_). However, the *r*_*i*_’s were not randomly distributed over space and season. So, to avoid biasing *r*_*lake*_ toward the season or location in space with the most *r*_*i*_’s, we used boosted regression trees to factor out those biases. To do this, we fit boosted regression trees separately for each lake which modelled the observed *r*_*i*_’s as a function of latitude, longitude, and day of the year with a total of *n* observations (*r*_*i*_’s). Boosted regression trees were used because they allow for nonlinear relationships between independent and dependent variables and high levels of interactions among independent variables (e.g. the effect of season could depend nonlinearly on latitude and longitude simultaneously). To reduce the computation intensity of this work, models were fit to a random subset of *n* = 10,000 observations for lakes which had more than 10,000 *r*_*i*_’s (n = 10,000 for 11 lakes, see Supplementary Table 1). We used each lake’s boosted regression trees to generate *n* modelled *r*_*i*_’s (${\hat{r}}_{i}$’s), using a randomly selected latitude-longitude combination and a randomly selected day of the year from the lists of unique latitude-longitude combinations and unique days of the year with at least one observed correlation coefficient. To each $\hat{r}i$, we added a randomly selected residual (*e*_*i*_) from the distribution of residuals from the boosted regression trees without replacement. *r*_*lake*_ was calculated as the mean $(\hat{r}i+{e}_{i})$ for each lake. We used non-parametric Wilcox tests to test whether *r*_*lake*_ was significantly different from zero.

To determine which lake characteristics were most strongly associated with *r*_*lake*_, we used boosted regression trees with elevation, latitude, lake surface area, lake perimeter, mean depth, salinity, median temperature, and median chl-a as predictors of *r*_*lake*_. Boosted regression trees were used here as well because they allow for nonlinear relationships between independent and dependent variables and high levels of interactions among independent variables. Except for salinity, the predictor variables used were from the attribute table of the GLWD-1 database. Salinity information was gathered from published literature and was represented in the model as a categorical variable (fresh or saline) following definitions from published literature^53^. We had an incomplete predictor matrix with some missing data (see Supplementary Information), but boosted regression trees can accommodate partially missing data without dropping observations from the model. We weighted each *r*_*lake*_ in the model by the significance of its p-value (observation weight = 1-p value) so that less significant correlations had less influence on the model outcome. To visualize the effect of median chl-a alone on the correlation between chl-a and LST, we removed variability attributable to all other predictor variables (black line in Fig. 3).

All boosted regression trees were fit with a model complexity value which matched the number of predictors in each model. The learning rates for the boosted regression trees in our study were optimized such that the final models included at least 1,000 trees but not more than 10,000 trees following recommendations from published literature^54^. Lakes from the GLWD dataset for which there were not sufficient data to fit boosted regression trees with a learning rate of at least 0.0001 were eliminated from our analyses. All boosted regression trees^54^ were fit using the ‘dismo’ package in the R environment for statistical computing^55^.

### Data Validation

We validated the chl-a and LST data using *in situ* data collected by the Environmental Protection Agency (EPA) from the North American Great Lakes. *In situ* data used here were collected in the North American Great Lakes primarily during two annual field campaigns in early and late summer from 2002–2016. We merged the EPA’s *in situ* data with the nearest coincident satellite data and directly compared them using standard major axis regression (SMA). We found that for all lakes, *in situ* chl-a and LST were significantly correlated to satellite-based chl-a and LST (Pearson correlation, p < 0.01). R^2^ from the SMA for the relationship between *in situ* and satellite chl-a and LST were 0.78 and 0.99, respectively. The median absolute error for chl-a and LST were 0.26 mg m^-3^ and 0.44 °C, respectively. The slopes in the SMAs for chl-a varied from 0.66 (Lake Erie) to 1.23 (Lake Ontario) across the North American Great Lakes due to differences in their optical conditions (geographical, atmospheric, and aquatic). The slopes in the SMAs comparing remotely-sensed to *in situ* LST varied from 0.98 (Lake Ontario) to 1.06 (Lake Superior) across the North American Great Lakes (See Supplementary Table 1 for full statistics table).

We also validated the lake-wide median chl-a and the lake-wide correlation coefficients between chl-a and LST using the *in situ* EPA data from the North American Great Lakes. Median lake chl-a from *in situ* and remote sensing data were highly correlated (Pearson correlation, r = 0.98, p < 0.01) with an SMA slope not significantly different from 1 (n = 5, slope = 0.89, 95% CI =  +/− 0.17,). Lake-wide correlations between chl-a and LST were also highly correlated (Pearson correlation, n = 5 r = 0.89, p = 0.02) with an SMA slope not significantly different from 1 (slope = 1.117 95%, confidence interval =  +/− 0.49,). These results show that even if the relationship between *in situ* and remotely sensed chl-a is at times weak (see Lake Superior in Supplementary Table 1), the lake-wide correlation coefficients are a robust indicator of the directionality of chl-a responses to LST (See Supplementary Table 1 for full statistics table). All figures were made using the R computing environment^55^ using the package, ‘ggplot2’^56^.

### Data Availability

All satellite-derived LST and chl-a datasets analysed during the current study are publicly available through NASA’s Physical Oceanography Distributed Active Archive Center (https://podaac.jpl.nasa.gov/). *In situ* temperature and chl-a data analysed in the current study are available through the United States Environmental Protection Agency (EPA) Central Data Exchange (https://cdx.epa.gov/). Lake polygons and characteristics are available through the Global Lakes and Wetlands Database (https://www.worldwildlife.org/pages/global-lakes-and-wetlands-database).

## Electronic supplementary material

Supplementary Information
