# Supplementary material for: Reconciling the opposing effects of warming on phytoplankton biomass in 188 large lakes
Source: Sci Rep. 2017 Sep 7;7:10762. doi: 10.1038/s41598-017-11167-3 (PMC5589843; doi:10.1038/s41598-017-11167-3)
Supplement: Supplementary file 1 — Supplementary Information [file 41598_2017_11167_MOESM1_ESM.doc]

**Supplementary Material**

Title: Reconciling the opposing effects of warming on phytoplankton biomass in 188 large lakes

Authors: Benjamin M. Kraemer*^1^, Thomas Mehner^1^, Rita Adrian^1^

1. IGB Leibniz-Institute of Freshwater Ecology and Inland Fisheries, Berlin, Germany, 12587

*Corresponding author: Benjamin Kraemer, bkraemer@igb-berlin.de, ben.m.kraemer@gmail.com

Supplementary Figure 1: In situ LST compared to remotely-sensed LST (GHRSST) for the North American Great Lakes from 2002-2016.

Supplementary Figure 2: In situ chl-a compared to remotely-sensed chl-a (MODIS-Aqua) for the North American Great Lakes from 2002-2016. The x and y axes are log-transformed to aid visualization of the data at low chl-a. Lines represent the least squares regressions of MODIS chl-a as a function of in situ chl-a for each lake. The wider scatter of the chl-a data at higher chl-a in Supplementary Figure 2 is consistent with higher scatter at higher chl-a for similar validation plots using ocean data over similar chl-a ranges (see https://seabass.gsfc.nasa.gov/search/?search_type=Perform%20Validation%20Search&val_sata=1&val_products=6).

Supplementary Figure 3: Lake-wide interannual correlation coefficient between chl-a and LST based on in situ (EPA) and remote sensing data (GHRSST, MODIS). Data are shown separately for each of the main field sampling seasons carried out by the EPA in early (Season 1) and late summer (Season 2).

Supplementary Table 1: Correlation coefficients and lake characteristics for each of the 188 lakes in our analyses.

| Lake name | Latitude | Longitude | Number (n) of correlation coefficients (r_i_) | Lake-wide LST and chl-a correlation (r_lake_) | Correlation p value | Lake-wide chl-a (mg m^3) |
| --- | --- | --- | --- | --- | --- | --- |
| Achit | 49.5 | 90.6 | 139 | 0.14 | <0.001 | 14.09 |
| Airag | 48.9 | 93.45 | 68 | 0.20 | 0.010 | 16.10 |
| Alakol | 46.1 | 81.7 | 1533 | -0.14 | <0.001 | 2.16 |
| Albert | 1.6 | 30.9 | 1362 | 0.10 | <0.001 | 31.25 |
| Amadjuak | 64.9 | -71.2 | 519 | -0.19 | <0.001 | 1.07 |
| Ang_tzu | 31 | 87.1 | 188 | 0.03 | 0.596 | 14.96 |
| Aqqikkol | 37.1 | 88.4 | 48 | 0.00 | 0.727 | 2.29 |
| Aral_Sea | 45.7 | 58.9 | 4885 | -0.12 | <0.001 | 6.97 |
| Argyle | -16.3 | 128.7 | 221 | -0.01 | 0.746 | 11.33 |
| Athabasca | 59.2 | -109.4 | 2920 | 0.02 | <0.001 | 9.89 |
| Ayakkum | 37.6 | 89.4 | 411 | -0.15 | <0.001 | 2.43 |
| Bahr_al_Milh | 32.7 | 43.6 | 62 | 0.16 | 0.170 | 23.39 |
| Baikal | 53.2 | 107.8 | 10000 | -0.04 | <0.001 | 2.82 |
| Baker | 64.2 | -95.4 | 197 | 0.14 | <0.001 | 28.56 |
| Balaton | 46.9 | 18 | 106 | -0.06 | 0.226 | 4.74 |
| Balkhash | 46.5 | 77.6 | 1875 | -0.05 | <0.001 | 2.08 |
| Bangong | 33.5 | 79.8 | 58 | -0.22 | <0.001 | 1.84 |
| Bangweulu | -11.2 | 29.8 | 863 | 0.07 | <0.001 | 21.19 |
| Becharof | 57.9 | -156.5 | 377 | 0.00 | 0.028 | 0.78 |
| Beloye | 60.2 | 37.6 | 354 | 0.01 | 0.556 | 27.51 |
| Beysehir | 37.7 | 31.5 | 271 | 0.20 | <0.001 | 3.54 |
| Biwa_ko | 35.3 | 136.1 | 99 | -0.06 | 0.001 | 7.48 |
| Blommesteinmeer_WJ_Van | 4.9 | -55.1 | 129 | -0.05 | 0.149 | 19.44 |
| Bodensee | 47.6 | 9.5 | 196 | -0.10 | 0.001 | 12.53 |
| Boeng_Tonle_Chhma | 12.9 | 104 | 432 | -0.13 | <0.001 | 11.54 |
| Boon_Tsagaan | 45.6 | 99.1 | 79 | 0.04 | 0.396 | 29.49 |
| Bosten | 42 | 87 | 442 | 0.02 | 0.275 | 16.05 |
| Bras_D_Or | 45.9 | -60.8 | 181 | 0.20 | <0.001 | 17.74 |
| Bratskoye | 56.05 | 101.9 | 124 | 0.03 | 0.126 | 34.40 |
| Buenos_Aires | -46.5 | -71.6 | 1119 | 0.05 | <0.001 | 0.38 |
| Buhayrat_ath_Tharthar | 33.9 | 43.3 | 1055 | -0.01 | 0.652 | 1.38 |
| Buyr | 47.8 | 117.7 | 177 | 0.03 | 0.065 | 33.28 |
| Cabora_Bassa | -15.7 | 32.1 | 677 | 0.21 | <0.001 | 13.11 |
| Cardiel | -48.9 | -71.2 | 172 | -0.17 | <0.001 | 2.19 |
| Caroni | 7.5 | -62.9 | 333 | 0.05 | 0.071 | 14.30 |
| Caspian_Sea | 41.2 | 50.7 | 10000 | 0.05 | <0.001 | 2.46 |
| Cedar | 53.2 | -100.1 | 391 | 0.17 | <0.001 | 19.41 |
| Cha_jih_nan_mu_tso | 30.9 | 85.6 | 374 | 0.34 | <0.001 | 1.87 |
| Chany | 54.9 | 77.9 | 88 | -0.03 | 0.272 | 8.90 |
| Chardarinskoye | 41.1 | 68.1 | 256 | 0.07 | <0.001 | 20.44 |
| Chiquita | -30.7 | -62.6 | 1065 | 0.18 | <0.001 | 35.63 |
| Churchill | 56 | -108.3 | 69 | 0.24 | <0.001 | 42.12 |
| Clearwater | 54.1 | -101.1 | 113 | 0.00 | 0.776 | 3.53 |
| Cold | 54.6 | -110.1 | 48 | -0.05 | 0.950 | 31.62 |
| Colville | 67.2 | -126 | 65 | 0.00 | 0.849 | 41.81 |
| Dead_Sea | 31.5 | 35.5 | 377 | 0.04 | 0.129 | 2.67 |
| Dore | 54.8 | -107.3 | 125 | 0.03 | 0.179 | 29.06 |
| Dorgon | 47.6 | 93.5 | 204 | 0.04 | 0.118 | 4.93 |
| Dubawnt | 63.1 | -101.5 | 323 | 0.18 | <0.001 | 15.81 |
| Edward | -0.4 | 29.6 | 354 | 0.11 | <0.001 | 24.07 |
| Egridir | 38 | 30.9 | 210 | 0.27 | <0.001 | 2.90 |
| Fagnano | -54.6 | -67.7 | 90 | 0.12 | <0.001 | 0.98 |
| Flathead | 47.9 | -114.1 | 140 | -0.06 | 0.262 | 10.06 |
| Garda | 45.5 | 10.6 | 113 | 0.20 | <0.001 | 3.50 |
| Geneva | 46.4 | 6.6 | 369 | 0.01 | 0.227 | 8.67 |
| Gozha | 35 | 81.05 | 48 | -0.18 | <0.001 | 0.55 |
| Great_Bear | 66 | -120.9 | 10000 | 0.01 | 0.239 | 1.79 |
| Great_Salt | 41.1 | -112.5 | 1033 | -0.12 | <0.001 | 9.38 |
| Great_Slave | 61.6 | -114.4 | 8883 | 0.03 | <0.001 | 7.64 |
| Habbaniyah | 33.3 | 43.5 | 103 | 0.07 | 0.920 | 5.98 |
| Har | 48 | 93.3 | 559 | 0.12 | <0.001 | 3.08 |
| Har_us | 48 | 92.3 | 264 | 0.11 | <0.001 | 19.41 |
| Hazen | 81.8 | -71.6 | 85 | 0.13 | <0.001 | 0.49 |
| Hottah | 65.2 | -118.6 | 60 | -0.02 | 0.957 | 21.58 |
| Hovs_Gol | 51.1 | 100.5 | 974 | -0.04 | <0.001 | 0.32 |
| Hsu_ju | 30.3 | 86.4 | 83 | 0.42 | <0.001 | 0.46 |
| Hyargas | 49.2 | 93.2 | 890 | -0.09 | <0.001 | 4.25 |
| Ihotry | -21.9 | 43.7 | 50 | -0.08 | 0.058 | 19.90 |
| Ijsselmeer | 52.6 | 5.3 | 367 | -0.01 | 0.240 | 28.22 |
| Iliamna | 59.5 | -155.2 | 1016 | 0.06 | <0.001 | 1.76 |
| Issyk_kul | 42.4 | 77.3 | 5048 | 0.06 | <0.001 | 0.31 |
| Izabal | 15.5 | -89.2 | 336 | 0.20 | <0.001 | 17.02 |
| Iznik | 40.4 | 29.5 | 160 | -0.03 | 0.886 | 22.21 |
| Jili | 46.9 | 87.4 | 74 | 0.29 | <0.001 | 13.55 |
| Kakhovskoye | 47.5 | 35 | 173 | 0.17 | <0.001 | 35.66 |
| Kakisa | 60.9 | -117.7 | 78 | 0.06 | 0.048 | 14.97 |
| KapchagayskoyeVodo | 43.8 | 77.5 | 783 | -0.02 | 0.701 | 4.05 |
| Kara_Bogaz_Gol | 41.2 | 53.6 | 1181 | 0.10 | <0.001 | 1.00 |
| Kariba | -16.8 | 28.3 | 1844 | 0.08 | <0.001 | 30.84 |
| Kayrakkumskoye | 40.3 | 69.9 | 195 | 0.07 | 0.004 | 6.76 |
| Khantayskoye | 68.3 | 90.1 | 48 | 0.04 | 0.105 | 30.77 |
| Kivu | -2 | 29.1 | 111 | -0.06 | <0.001 | 7.64 |
| Kiziliashskiy | 45.1 | 37.1 | 69 | 0.15 | <0.001 | 10.90 |
| Kotelny | 75.6 | 142.5 | 52 | 0.00 | 0.584 | 5.83 |
| Kremenshugskoye | 49.3 | 32.6 | 82 | -0.04 | 0.095 | 35.86 |
| Kulundinskoye | 53 | 79.5 | 286 | -0.31 | <0.001 | 15.30 |
| Kuybyshevskoye | 55.2 | 49.4 | 717 | 0.08 | <0.001 | 32.78 |
| Kuyumazarskoye | 39.9 | 64.8 | 149 | -0.02 | 0.259 | 10.29 |
| Kyoga | 1.5 | 32.7 | 312 | 0.07 | <0.001 | 47.49 |
| Labaz | 72.3 | 99.6 | 134 | 0.24 | <0.001 | 7.12 |
| Ladoga | 60.7 | 31.6 | 3692 | 0.08 | <0.001 | 16.80 |
| Erie | 42.1 | -81.3 | 10000 | 0.12 | <0.001 | 3.46 |
| Huron | 45 | -82.4 | 10000 | -0.15 | <0.001 | 0.68 |
| Marion | 33.5 | -80.3 | 73 | -0.18 | 0.003 | 20.03 |
| Maurepas | 30.2 | -90.5 | 78 | 0.10 | 0.028 | 24.55 |
| Michigan | 43.9 | -86.9 | 10000 | -0.10 | <0.001 | 0.83 |
| Moultrie | 33.3 | -80.1 | 57 | -0.02 | 0.410 | 26.54 |
| Lake_of_the_Woods | 49 | -95 | 309 | 0.04 | 0.006 | 30.21 |
| Okeechobee | 26.9 | -80.8 | 65 | -0.11 | 0.231 | 40.60 |
| Ontario | 43.6 | -77.8 | 10000 | 0.08 | <0.001 | 1.88 |
| Saint_Clair | 42.4 | -82.7 | 366 | -0.05 | <0.001 | 3.02 |
| Superior | 47.5 | -87.6 | 10000 | -0.01 | <0.001 | 0.90 |
| Tahoe | 39.1 | -120.1 | 406 | -0.06 | 0.001 | 0.25 |
| Victoria | -1 | 33.1 | 10000 | 0.01 | 0.003 | 25.62 |
| Lama | 69.4 | 89.3 | 101 | 0.06 | 0.266 | 8.76 |
| Lianquihui | -41.1 | -72.8 | 302 | -0.07 | <0.001 | 0.29 |
| Limfjorden | 56.9 | 9.1 | 95 | 0.17 | <0.001 | 32.58 |
| Lumajangdong | 34 | 81.6 | 70 | 0.32 | <0.001 | 0.44 |
| Ma_pang_yung_tso | 30.7 | 81.4 | 140 | -0.16 | <0.001 | 1.19 |
| Madre | 25.1 | -97.7 | 136 | -0.04 | 0.021 | 3.85 |
| Malawi | -12 | 34.5 | 10000 | -0.19 | <0.001 | 0.67 |
| Malombe | -14.7 | 35.2 | 139 | 0.25 | <0.001 | 24.32 |
| Managua | 12.3 | -86.4 | 101 | 0.34 | <0.001 | 6.88 |
| Mangueira | -33.1 | -52.8 | 177 | 0.01 | 0.401 | 14.56 |
| Manitoba | 50.7 | -98.5 | 1295 | 0.14 | <0.001 | 12.68 |
| Markakol | 48.8 | 85.7 | 239 | 0.16 | <0.001 | 20.39 |
| Martre | 63.4 | -118 | 438 | -0.05 | 0.007 | 24.57 |
| Maud | 67.8 | -97.6 | 108 | 0.02 | 0.335 | 1.96 |
| Mille_Lacs | 46.2 | -93.7 | 105 | 0.11 | 0.001 | 38.28 |
| Mingechaurskoye | 40.9 | 46.7 | 280 | 0.26 | <0.001 | 7.59 |
| Musters | -45.5 | -69.2 | 131 | -0.11 | <0.001 | 9.83 |
| Mweru | -9 | 28.7 | 1770 | -0.02 | 0.034 | 41.30 |
| Na_Mu | 30.7 | 90.6 | 933 | 0.25 | <0.001 | 0.55 |
| Nasser | 23 | 32.6 | 929 | -0.16 | <0.001 | 23.51 |
| Ngoring | 34.9 | 97.7 | 139 | -0.18 | <0.001 | 3.05 |
| Nicaragua | 11.5 | -85.4 | 2134 | 0.07 | <0.001 | 17.41 |
| Nipigon | 49.8 | -88.5 | 372 | 0.00 | 0.905 | 24.94 |
| Nipissing | 46.3 | -79.7 | 89 | -0.07 | 0.012 | 25.94 |
| North_Moose | 54.1 | -100.2 | 58 | 0.04 | 0.496 | 8.81 |
| Novosibirskoye | 54.7 | 82.8 | 198 | 0.11 | <0.001 | 21.56 |
| Ohrid | 41 | 20.7 | 187 | 0.00 | 0.487 | 0.66 |
| Onegh | 61.6 | 35.6 | 112 | 0.09 | <0.001 | 0.56 |
| Orog | 50.1 | 91 | 76 | 0.28 | <0.001 | 1.99 |
| Pei_ku_tso | 28.9 | 85.6 | 88 | -0.05 | 0.399 | 0.61 |
| Peipus | 58.7 | 27.5 | 812 | 0.10 | <0.001 | 45.68 |
| Pervoye | 67.9 | 71.6 | 79 | 0.07 | 0.348 | 26.14 |
| Primrose | 54.9 | -109.7 | 53 | -0.07 | 0.380 | 26.10 |
| Pyasino | 70 | 87.8 | 103 | 0.21 | <0.001 | 6.07 |
| Pyramid | 40.1 | -119.6 | 205 | -0.04 | 0.018 | 2.20 |
| Qinghai | 36.9 | 100.2 | 1685 | -0.02 | 0.003 | 2.20 |
| Red_Upper_and_Lower | 48 | -94.9 | 500 | 0.11 | <0.001 | 34.44 |
| Reindeer | 57.6 | -102.2 | 275 | 0.00 | 0.322 | 19.94 |
| Ronge | 55 | -105.1 | 111 | 0.17 | <0.001 | 31.49 |
| Rybinkskoye | 58.4 | 38.45 | 232 | 0.20 | <0.001 | 34.05 |
| Sai_li_mu | 44.6 | 81.2 | 245 | 0.03 | 0.018 | 0.43 |
| Saitlan | 55 | 78.6 | 109 | -0.15 | 0.007 | 15.73 |
| Salton_Sea | 33.3 | -115.8 | 360 | 0.03 | 0.137 | 37.51 |
| Sarygamysh | 41.9 | 57.4 | 188 | -0.06 | 0.093 | 2.27 |
| Sarykamyshskoye | 41.9 | 57.5 | 124 | -0.05 | 0.003 | 2.27 |
| Scutari | 42.2 | 19.3 | 291 | 0.14 | <0.001 | 8.07 |
| Se_lin | 31.8 | 89 | 695 | -0.43 | <0.001 | 0.75 |
| Selawik | 66.5 | -160.7 | 391 | 0.06 | <0.001 | 25.37 |
| Sevan | 40.3 | 45.4 | 928 | 0.21 | <0.001 | 5.42 |
| Shalkar | 50.6 | 51.7 | 124 | -0.17 | <0.001 | 30.19 |
| Shishmaref | 66.2 | -165.9 | 81 | 0.17 | <0.001 | 13.98 |
| Simcoe | 44.4 | -79.4 | 170 | 0.02 | 0.801 | 24.43 |
| Smallwood | 54 | -64.1 | 173 | 0.08 | <0.001 | 10.77 |
| Solenoye | 45.4 | 35.1 | 91 | 0.01 | 0.940 | 14.49 |
| Song_kel | 41.85 | 75.1 | 108 | 0.04 | 0.082 | 2.95 |
| Southern_Indian | 57.4 | -98.3 | 84 | -0.35 | <0.001 | 8.82 |
| St_Martin | 51.7 | -98.5 | 70 | -0.04 | 0.867 | 18.59 |
| St_Peter | 46.2 | -72.9 | 96 | 0.14 | <0.001 | 8.88 |
| Ta_jo | 31.1 | 84.1 | 217 | 0.28 | <0.001 | 0.90 |
| Tamiahua | 21.6 | -97.6 | 143 | -0.05 | 0.005 | 8.46 |
| Tang_je_yung_tso | 31.1 | 86.7 | 301 | 0.15 | <0.001 | 0.56 |
| Tanganyika | -6.7 | 29.8 | 10000 | -0.14 | <0.001 | 0.76 |
| Tapajos | -2.4 | -55 | 244 | 0.06 | 0.001 | 17.75 |
| Taupo | -38.8 | 175.9 | 294 | -0.03 | 0.012 | 1.17 |
| Taymyr | 74.6 | 101.8 | 699 | 0.21 | <0.001 | 12.47 |
| Telmen | 48.9 | 97.3 | 83 | 0.08 | 0.003 | 18.46 |
| Tengiz | 50.4 | 68.9 | 316 | 0.01 | 0.299 | 10.76 |
| Teshekpuk | 70.6 | -153.6 | 132 | 0.20 | <0.001 | 6.86 |
| Titicaca | -15.9 | -69.3 | 4326 | -0.08 | <0.001 | 2.48 |
| Tsimlyanskoye | 47.8 | 42.8 | 457 | 0.08 | <0.001 | 44.00 |
| Tulemalu | 62.9 | -99.4 | 117 | 0.12 | <0.001 | 11.17 |
| Turkana | 3 | 36.3 | 1537 | 0.05 | <0.001 | 9.26 |
| Ulungar | 47.3 | 87.3 | 444 | 0.12 | <0.001 | 5.15 |
| Urmia | 37.9 | 45.3 | 1375 | 0.15 | <0.001 | 2.33 |
| Uvs | 50.3 | 92.7 | 1671 | -0.15 | <0.001 | 2.37 |
| Van | 38.6 | 42.9 | 2436 | -0.07 | <0.001 | 1.19 |
| Vanern | 58.9 | 13.3 | 3236 | 0.13 | <0.001 | 20.39 |
| Vattern | 58.4 | 14.5 | 953 | 0.10 | <0.001 | 16.74 |
| Villarrica | -39.3 | -72.1 | 113 | -0.13 | 0.001 | 12.74 |
| Williston | 56 | -123.9 | 89 | 0.21 | 0.004 | 16.44 |
| Winnipeg | 52.9 | -98.1 | 7882 | 0.15 | <0.001 | 11.26 |
| Winnipegosis | 52.7 | -100 | 1427 | 0.07 | <0.001 | 11.95 |
| Wollaston | 58.2 | -103.3 | 102 | -0.05 | 0.026 | 12.88 |
| Zaysan | 48.1 | 83.8 | 1077 | 0.00 | 0.081 | 15.11 |

Supplementary Table 2: Statistics table for standard major axis regression (SMA) analyses comparing in situ data to remote sensing data for chl-a, LST, lake-wide median chl-a, and the correlation coefficient between chl-a and LST. All comparisons were made using SMA with remote sensing values on the y axis and in situ values on the x axis. Remote-sensing and in situ data were compared across all lakes and separately for each lake as indicated in the “domain” column. The slope and intercept p values corresponds to tests of the null hypotheses that the slope = 1 and the intercept = 0.

| **Variable** | **Domain** | **Slope** | **± 95% CI** | **Slope p value** | **Intercept** | **± 95% CI** | **Intercept p value** | **R^2^** | **Correlation p value** | **n** |
| --- | --- | --- | --- | --- | --- | --- | --- | --- | --- | --- |
| chl-a | all lakes | 0.900 | 0.047 | <0.001 | -0.015 | 0.020 | 0.137 | 0.783 | <0.001 | 300 |
|  | Erie | 0.664 | 0.097 | <0.001 | 0.119 | 0.071 | 0.001 | 0.463 | <0.001 | 87 |
|  | Huron | 1.166 | 0.216 | 0.139 | 0.004 | 0.066 | 0.907 | 0.560 | <0.001 | 44 |
|  | Michigan | 0.978 | 0.179 | 0.829 | -0.073 | 0.032 | <0.001 | 0.613 | <0.001 | 40 |
|  | Ontario | 1.231 | 0.242 | 0.063 | -0.079 | 0.096 | 0.108 | 0.470 | <0.001 | 46 |
|  | Superior | 1.185 | 0.222 | 0.108 | 0.052 | 0.046 | 0.028 | 0.110 | 0.002 | 83 |
|  |  |  |  |  |  |  |  |  |  |  |
| LST | all lakes | 0.996 | 0.006 | 0.216 | 0.188 | 0.090 | <0.001 | 0.993 | <0.001 | 764 |
|  | Erie | 0.981 | 0.009 | <0.001 | 0.372 | 0.159 | <0.001 | 0.994 | <0.001 | 264 |
|  | Huron | 0.992 | 0.009 | 0.089 | 0.337 | 0.140 | <0.001 | 0.997 | <0.001 | 144 |
|  | Michigan | 0.998 | 0.015 | 0.835 | 0.115 | 0.244 | 0.350 | 0.995 | <0.001 | 88 |
|  | Ontario | 0.979 | 0.013 | 0.003 | 0.619 | 0.220 | <0.001 | 0.996 | <0.001 | 86 |
|  | Superior | 1.056 | 0.021 | <0.001 | -0.411 | 0.219 | <0.001 | 0.982 | <0.001 | 182 |
|  |  |  |  |  |  |  |  |  |  |  |
| Median chl-a | all lakes | 0.890 | 0.174 | 0.230 | -0.020 | 0.078 | 0.465 | 0.985 | 0.001 | 5 |
|  |  |  |  |  |  |  |  |  |  |  |
| LST chl-a Correlation | all lakes | 1.117 | 0.490 | 0.604 | 0.117 | 0.123 | 0.056 | 0.890 | 0.016 | 5 |
